# Genomewide DNA methylation dynamics upon diesel exhaust exposure in asthmatics

**DOI:** 10.1186/1710-1492-10-S1-A67

**Published:** 2014-03-03

**Authors:** Ruiwei Jiang, Francesco Sava, Michael S Kobor, Christopher R Carlsten

**Affiliations:** 1 Genome Sciences and Technology, College for Interdisciplinary Studies, University of British Columbia, Vancouver, British Columbia, Canada, V6T 1Z4; 2 Department of Medicine, Division of Respiratory Medicine, University of British Columbia, Vancouver, British Columbia, Canada, V5Z 1M9; 3 Department of Medical Genetics, University of British Columbia, Vancouver, British Columbia, Canada, V5Z 4H4

## Background

Particulate air pollution can induce epigenetic changes and regulate gene expression relevant to the pathophysiology of asthma and allergic diseases. Recently, epidemiologic data suggests that there are observable acute effects of air pollution on peripheral blood DNA methylation levels of genomewide Alu and LINE-1 repeat elements, as well as certain genes involved in oxidative stress response and innate immunity. In this study, we hypothesized that in a controlled exposure setting, diesel exhaust (as a model of particulate air pollution) can induce DNA methylation changes that are detectable on the genomewide level.

## Methods

We recruited 16 subjects with asthma, and/or airway hyper-responsiveness. They were exposed to both diesel exhaust (DE) and filtered air (FA) following a randomized crossover design. Peripheral blood mononuclear cells (PBMCs) were collected at baseline, 6 hours, and 30 hours post-exposure. Methylation at 415,382 CpG sites covering 39,136 genes was measured using the Illumina Infinium 450K bead chip methylation array. To detect effects of the diesel exposure, we conducted a principal component analysis (PCA) , resulting in principal components with common patterns of methylation variation across samples. Using this method we were able to pinpoint one principal component that was significantly associated with diesel exhaust exposure, from which we then selected a subset of probes that possessed that specific pattern of variation.

## Results

**Figure 1:**
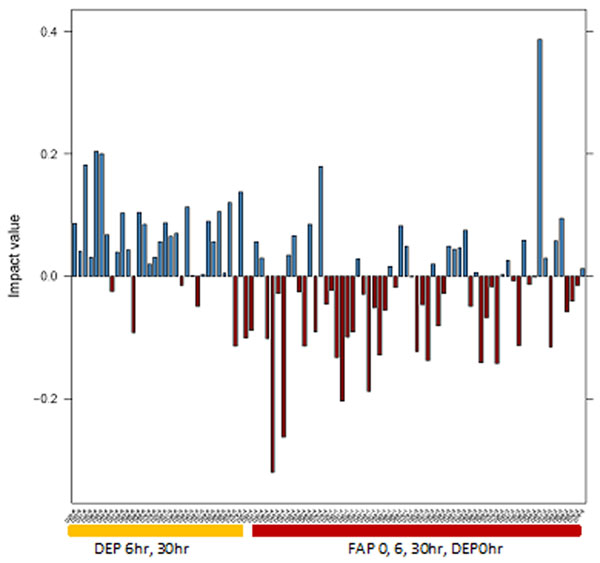
Impact values of each sample in principal component 22. Samples were separated into DEP 6hr and 30hr, followed by FAP 0hr, 6hr and 30hr, and DEP 0hr.

**Figure 2:**
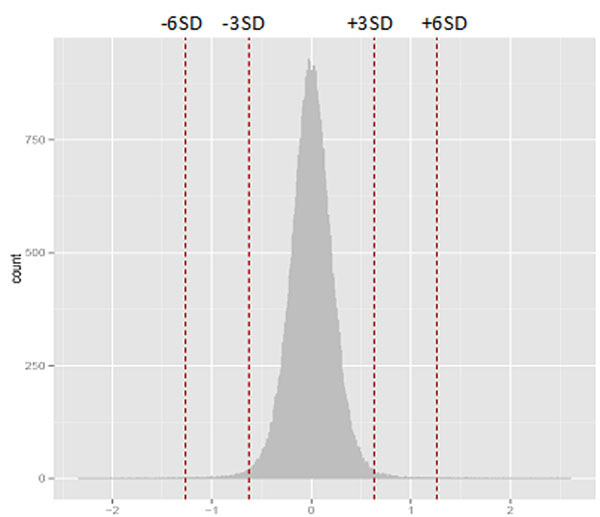
Distribution of probe loading values for principal component 22. The values located at ±3 and ±6 standard deviations are marked.

Whole genome analysis using PCA followed by denoising revealed that principal component 22, which accounted for 0.5% of the total variance, was significantly associated with the treatment variable: [DE 6hr and 30hr] versus [DE 0hr, FA 0hr, 6hr, and 30hr] (Figure 1). Using loading cutoff of ±6 standard deviations, we found 89 CpG sites to possess the specific pattern of variation (Figure 2). These include genes whose expression is associated with exposure to either diesel exhaust or components of diesel exhaust as reported by literature: CASP7, ATCAY, ABCA1, JAK3, CYFIP2, and NOX2 [1-6].

## Conclusions

These results suggest that short-term exposure to diesel exhaust in a controlled setting has minimal but detectable effects on a genomewide level in PBMCs. We are currently applying mixed effects modeling and intraclass correlation to our identified hits to further substantiate the association of these hit probes to the treatment variable.
